# A dynamic anthropomorphic phantom for end‐to‐end testing in image‐ and surface‐guided adaptive radiotherapy

**DOI:** 10.1002/mp.70107

**Published:** 2025-11-03

**Authors:** Anahita Bakhtiari Moghaddam, A. Runz, R. Figueiredo Augusto, G. Echner, W. Johnen, R. Gabriel, P. Haering, C. Lang, S. Seeber, C. Murillo, B. Ackermann, R. Pestana, C. Beyer, F. Weykamp, M. Jochim, A. Qubala, V. Batista, O. Jäkel, C. P. Karger

**Affiliations:** ^1^ Faculty of Medicine University of Heidelberg Heidelberg Germany; ^2^ Division of Medical Physics in Radiation Oncology German Cancer Research Center (DKFZ) Heidelberg Germany; ^3^ Heidelberg Institute for Radiation Oncology (HIRO) National Center for Radiation Research in Oncology (NCRO) Heidelberg Germany; ^4^ Division of Medical Physics in Radiology German Cancer Research Center (DKFZ) Heidelberg Germany; ^5^ Heidelberg Ion Beam Therapy Center (HIT) University Hospital Heidelberg Heidelberg Germany; ^6^ Department of Radiation Oncology Heidelberg University Hospital Heidelberg Germany; ^7^ Clinical Cooperation Unit Radiation Oncology German Cancer Research Center (DKFZ) Heidelberg Germany; ^8^ Division of Radiology German Cancer Research Center (DKFZ) Heidelberg Germany

**Keywords:** adaptive radiotherapy, anthropomorphic phantom, end to end test

## Abstract

**Background:**

Respiratory and digestive motion can compromise the accuracy of radiotherapy (RT), affecting tumor targeting and healthy tissue sparing. Current phantoms often fail to replicate complex organ movements and lack compatibility with advanced imaging modalities like magnetic resonance imaging (MRI) and 4D computed tomography (4DCT), limiting their utility in adaptive radiotherapy. The BRaVIDA (Breathing Radiotherapy Visual monitoring, Imaging, and Dosimetric Anthropomorphic) phantom addresses these limitations by simulating realistic organ motion during breathing and digestion. Its MRI compatibility allows for comprehensive testing in adaptive radiotherapy workflows, improving motion management and treatment precision for both photon and ion beam therapies.

**Purpose:**

This study introduces the **BRaVIDA** (**B**reathing **Ra**diotherapy **V**isual monitoring, **I**maging, and **D**osimetric **A**nthropomorphic) phantom, a novel anthropomorphic phantom designed to simulate respiratory motion and support end‐to‐end testing in adaptive radiotherapy. The aim is to improve motion management, imaging accuracy, and dosimetric validation for radiotherapy treatments.

**Methods:**

The BRaVIDA phantom is designed with realistic anatomical structures of the thorax and abdomen and may be equipped with dosimeters. It simulates respiratory and digestive motion using an in‐house developed hydraulic system. The phantom was imaged by CT and MRI, using different protocols (CT, MRI, 4DCT, and 4DMRI) to evaluate image contrast and motion parameters. Dosimetric properties were assessed by measuring the relative electron density (RED) and the stopping power ratio (SPR) of ion beams and the results were compared with the values of the treatment planning system (TPS).

**Results:**

The phantom demonstrated realistic image contrast in CT and MRI, with anthropomorphic characteristics similar to human tissues. Motion amplitudes for various organ models (pancreas, stomach, liver) were successfully measured using 4DCT and 4DMRI. Dosimetric testing showed that the RED and SPR values of BRaVIDA align closely with TPS reference values. Deviations in photon attenuation were below 1.5% for all phantom materials.

**Conclusions:**

BRaVIDA presents a versatile, MRI‐compatible phantom exhibiting anthropomorphic image contrast, inter‐ and intrafractional motion as well as radiation attenuation in photon beams, and equipping the phantom with detectors allows for full end‐to‐end‐tests in adaptive image‐ and surface‐guided photon RT workflows without further adaptions. For ion beams, the SPR values in the TPS have to be adapted to the measured data to assure correct range calculation in the phantom. The phantom presents a valuable and accurate tool for clinical and research applications in adaptive RT.

## 1 INTRODUCTION

Respiratory‐induced motion in radiotherapy (RT) can lead to target displacement, causing image artifacts that may distort tumor trajectory assessment and compromise treatment accuracy. This motion may result in unintended dose distributions to both the target volume and adjacent organs at risk, including complex interplay effects.^1^, ^2^ Tumors located in the lung and liver are particularly susceptible, as they experience continuous respiratory motion during treatment, with the largest displacements occurring in the superior‐inferior (SI) direction. Studies have reported liver tumor displacements of up to 25 mm during normal respiration, and up to 55 mm with deep breathing.^3^, ^4^, ^5^ In response to these challenges posed by motion‐related uncertainties, Image‐Guided Radiation Therapy (IGRT) has become a widely accepted standard in clinical practice. When combined with Surface‐Guided Radiation Therapy (SGRT), IGRT enhances patient safety by providing real‐time tracking of tumor motion and ensuring more accurate treatment delivery without additional exposure by ionizing radiation.^6^, ^7^.

Moreover, online adaptive RT workflows involve a continuous loop of patient positioning, imaging, image co‐registration, treatment plan (TP) adaptation, and irradiation. Uncertainties can arise at any stage of this process, and their accumulation can be evaluated through end‐to‐end (E2E) tests specific to the workflow. These tests require the use of appropriate phantoms,^8^ which enable the assessment of both static and dynamic conditions, as well as the measurement of dose distributions, either pointwise, in 2D or 3D, in different irradiation modes (e.g., with or without gating). As a result, the availability of anthropomorphic phantoms is important, and recent research has focused on developing and improving these phantoms. Currently, both in‐house developed and commercially available phantoms are being utilized for these purposes.^9^, ^10^, ^11^, ^12^, ^13^, ^14^, ^15^, ^16^, ^17^, ^18^, ^19^ However, existing phantoms often lack anthropomorphic shape, motion, and deformability on the one hand, and operational precision on the other hand.^20^

The aim of this study is the introduction of a novel 4D phantom of the thorax and abdomen, called the **B**reathing **Ra**diotherapy **V**isual monitoring, **I**maging and **D**osimetric **A**nthropomorphic (**BRaVIDA**) Phantom. This phantom is anthropomorphic in terms of shape, image contrast in computed tomography (CT) and magnetic resonance imaging (MRI) as well as photon attenuation. Its design enables the evaluation of radiation treatment quality using radiation detectors, such as ionization chambers (IC) and radiochromic films. Additionally, it is capable of simulating respiratory motion, including tumor motion, and is suitable for applications in SGRT. BRaVIDA therefore bridges the gap left by its predecessors, combining anatomical structures, mobility, and adaptability for multimodal imaging and dosimetric purposes in RT, while also enabling exploration of the potential of SGRT for motion monitoring.

## 2 METHODS

### 2.1 Phantom design

**FIGURE 1:**
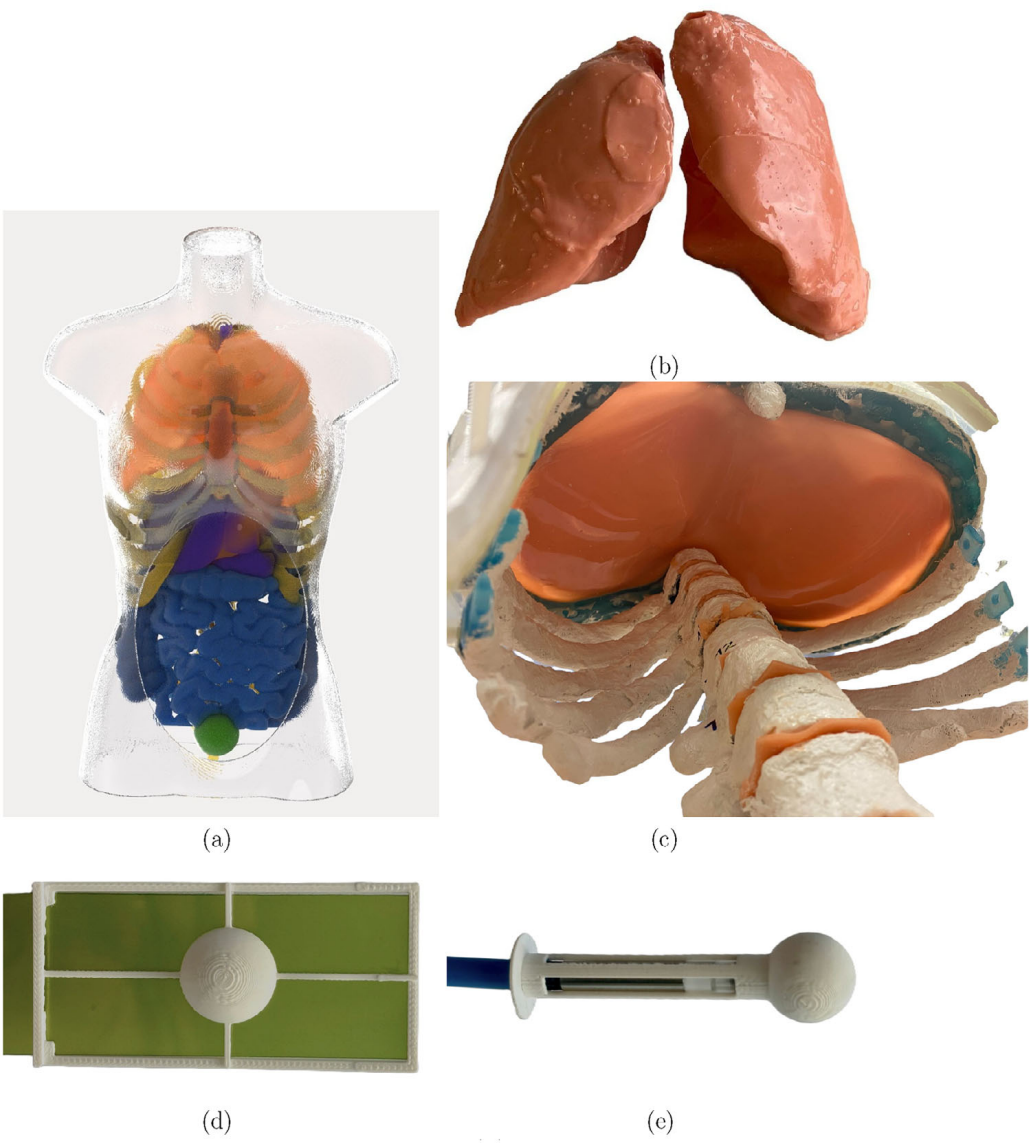
Schematic of BRaVIDA's composites. (a)The phantom's perimeters are: 98, 86, 73, and 90 cm for shoulders, thorax, waist, and hips. (b) Silicone lung model with hollow bronchi. (c) 3D‐printed model of the ribs, thoracic spine, and a 2mm layer of silicone representing the diaphragm fixed within the thorax region. (d) Abdomen tumor model with inserted film. (e) Liver tumor model with inserted Pinpoint ionization chamber (PTW, Freiburg, Germany).

To achieve realistic body proportions in a cost‐effective manner, a half‐body mannequin, size 38, was acquired and manufactured from transparent polystyrene plastic (Guangzhou Xinji Trading Co., Ltd., China). Subsequently, BRaVIDA was designed according to the shape of the mannequin (Figure 1).

The 3D‐printed bones were wrapped in a layer of gypsum (Vero‐Gyps (Bone)) to ensure that their Hounsfield units (HU) aligned with the respective values in patients. The thorax and abdomen regions were separated by a flexible diaphragm membrane made from TFC silicone type 6 with a shore hardness of 22 (Troll Factory Rainer Habekost e.K., Germany). The same silicone type was used for the abdominal wall and to cover the lower abdomen. In the abdominal cavity, the organ models were embedded within a flexible matrix (Techno GelGum, Raytech, Milan, Italy). This matrix ensures precise anatomical placement of the organs and allows organ movement during respiration.

### 2.2 Organ casting molds

Organ models were designed using data segmented and extracted from patient CT scans using the Medical Imaging Interaction Toolkit (MITK, DKFZ, Germany). Based on this segmentation, three‐dimensional casting molds for the organs were created using Inventor 2018 (Autodesk, USA), Meshmixer (Autodesk, USA), and Geomagic Freeform 2022 (3D Systems, USA). The molds were then filled with different materials, according to the anatomical and imaging characteristics of the organ (Table 1). Models for the liver, pancreas, spleen, and kidneys were produced by filling the 3D‐printed molds with a mixture of agarose, nickel‐diethylene triamine pentaacetic acid (NiDTPA), and potassium chloride (KCl), which provide anthropomorphic image contrast in CT and MRI, as described in by Elter et al.^21^ The lungs, stomach and colon were made from silicone type 6, the small intestine and duodenum from TFC silicone type 13 with a shore hardness of 0 (Troll Factory Rainer Habekost e.K., Germany), and the bladder from latex. The lungs were filled with air, while the colon, bladder and stomach were all filled with water. This design minimizes non‐anthropomorphic and unintended organ motion within the abdominal cavity.

**TABLE 1:** 3D organ models produced with casting molds and filling materials.

Organ models	Casting mold	Material
Liver	✓	Contrast gel
Pancreas	✓	Contrast gel
Kidney	✓	Contrast gel
Spleen	✓	Contrast gel
Small intestine	✓	Silicone T13
Duodenum	✓	Silicone T13
Lungs	✓	Silicone T6 (2mm) ‐ Air
Stomach	✓	Silicone T6 (2mm) ‐ Water
Colon	✓	Silicone T6 (2mm) ‐ Water
Bladder	✓	Latex (1mm) ‐ Water
Bones	—	VeroClear, Gypsum
Matrix	—	GelGum

### 2.3 Simulation of motion

**FIGURE 2:**
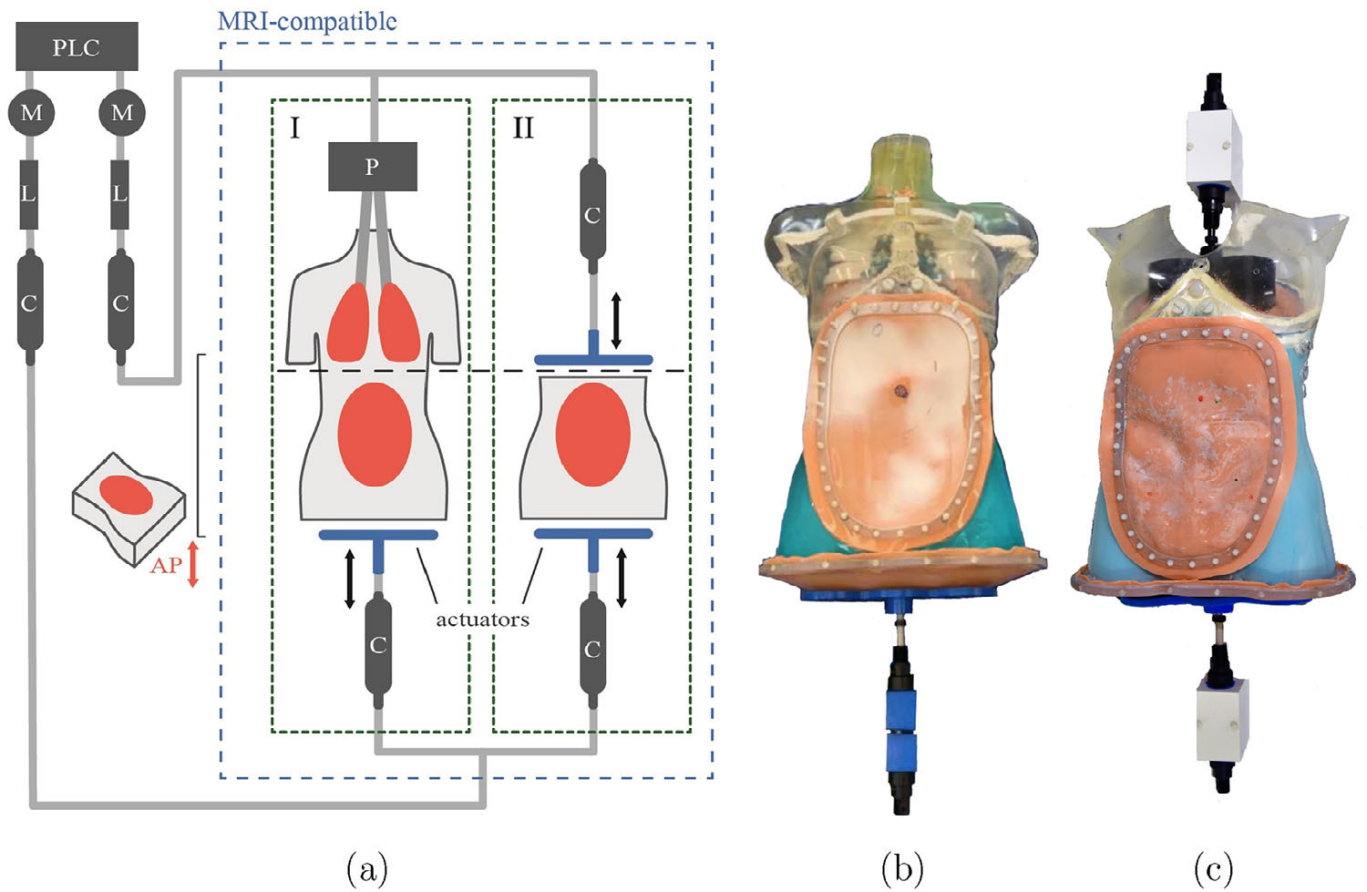
(a) Schematic of the two modes for generating motion in the phantom (dashed green boxes): (I) one actuator acts on the lower abdominal membrane while the lung is inflated and deflated, thereby indirectly acting on the diaphragm. (II) Two actuators act on the diaphragm and lower abdomen, respectively. The components in the dashed blue square are MRI‐compatible while the motors are located in the control room, operating the phantom via a 9‐meter oil‐filled tubing system. (b) shows the lung model with ribs and lower abdomen actuator in mode I, and (c) shows the diaphragm actuator and lower abdomen actuator in mode II.

The motion in the phantom is generated by an in‐house developed hydraulic system (HS) to simulate respiratory and digestive motion in the abdominal cavity using predefined motion patterns, controlled by an in‐house‐developed graphical user interface. The HS consists of two independent stepper motors (M), four MRI‐compatible double‐acting cylinders (C) (PSK Ingenieurgesellschaft mbH, Germany), and two linear actuators (L), all controlled by a Programmable Logic Controller (PLC) CX5020 (Beckhoff, Germany). The HS can be operated in two different modes (Figure 2) and both modes result in abdominal wall motion (AP motion).

1. **Indirect method (I)**: The lung model is inflated and deflated by a pneumatic system (P), which then indirectly moves the diaphragm, and an actuator on the lower abdominal membrane, thereby simulating respiratory motion in the phantom.
2. **Direct method (II)**: Two actuators are attached from two sides to the diaphragm and to the lower abdominal membrane to directly induce respiratory and/or digestive motion in the abdominal cavity.

### 2.4 Imaging

**FIGURE 3:**
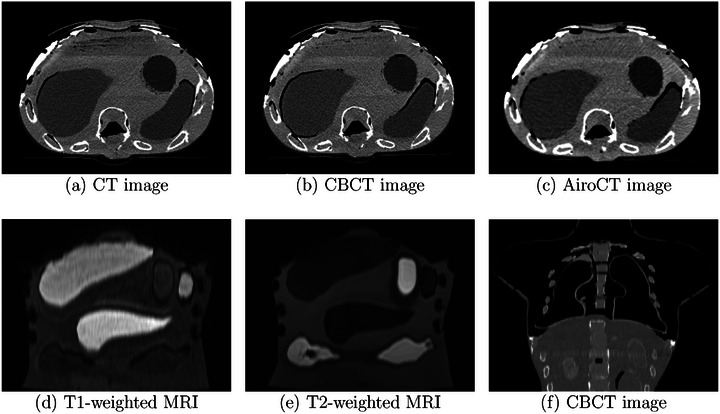
(a) CT image, (b) CBCT image from Ethos device, (c) AiroCT image, illustrating the phantom's anatomy and transversal images of the bones, liver, stomach, and spleen models. Coronal (d) T1‐weighted MRI image acquired with the VIBE Dixon sequence and (e) T2‐weighted MRI image acquired with the SPACE sequence showing the bones, liver, spleen, kidneys, pancreas, stomach, and sections of the colon models, (f) CBCT image of the lung region acquired with the Ethos device.

To evaluate contrast and motion pattern of the phantom, static and dynamic imaging protocols were employed for different imaging modalities (Figure 3).

#### 2.4.1 CT

The CT acquisition of the abdomen at different scanners was performed with the following parameters: (i) SOMATOM Definition Flash (Siemens, Erlangen, Germany) installed at German Cancer Research Center (DKFZ): 120 kV, 480 mA, voxel size (0.97 × 0.97 × 1) ${\mathrm{mm}}^{3}$; (ii) Mobile CT system (AiroCT, Mobius Medical, Houston, TX, USA) installed for positioning verification at the Heidelberg Ion‐Beam Therapy Center (HIT): 120 kV, 80 mA, voxel size (0.97 × 0.97 × 1) ${\mathrm{mm}}^{3}$; and (iii) Cone‐beam CT at and the ETHOS linear accelerator (Varian, Palo Alto, California, USA) installed at DKFZ: 140 kV, 90 mA, voxel size (1.36 × 1.36 × 2) ${\mathrm{mm}}^{3}$. The average HU of various organ models were measured in a region of interest (ROI) of abdomen using RadiAnt DICOM Viewer (Medixant, Version 2024.1).

#### 2.4.2 MRI

T1‐ and T2‐weighted images were acquired on a 1.5 T MRI (Magnetom Sola BioMatrix) at HIT. For T1‐weighted images, the T1 VIBE DIXON sequence was used to measure a transversal image, with a repetition time of 6.99 ms, an echo time of 2.39 ms and a voxel size of 1.04 × 1.04 × 3 ${\mathrm{mm}}^{3}$. For T2‐weighted images, a T2 SPACE sequence was employed to acquire a transversal image with a repetition time 1400 ms, an echo time of 134 ms and a pixel size of 1.04 × 1.04 × 3 ${\mathrm{mm}}^{3}$. Additionally, T1 and T2 relaxation times were measured using the in‐house developed software MITK v2024.10.

#### 2.4.3 Temporal resolved imaging

For 4D computed tomography (4DCT) and 4D magnetic resonance imaging (4DMRI), the phantom was operated in dynamic mode II (Figure 2), utilizing a respiratory motion model described by Lujan et al.^22^:

$$s\left(t\right)=s_{0}+A\;\cos^{2n}\left(\frac{\pi t}{\tau }+\phi \right)$$

where $s_{0}$ represents the tumor position during exhalation, A is the amplitude, τ is the breathing cycle period, ϕ is the initial phase, and n determines the shape of the curve. By adjusting these parameters or using recorded patient breathing patterns, the phantom can simulate regular or abitrary irregular motion patterns. The 4DCT and 4DMRI experiments were conducted on different days using the following settings, summarized in Table 2:

**TABLE 2:** 4DMRI and 4DCT experimental settings for diaphragm and pelvic actuators.

Modality	Actuator	$s_{0}$ (mm)	A (mm)	n	τ (s)	ϕ ( 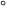 )
4DMRI	Diaphragm	8	30	1	7	90
	Pelvic	8	10	1	7	0
4DCT	Diaphragm	8	15	1	7	90
	Pelvic	8	5	1	7	0

Abbreviations: 4DCT, 4D computed tomography; 4DMRI, 4D magnetic resonance imaging.

The 4DCT was acquired on a SOMATOM Definition Flash by using an Anzai belt (Anzai Medical Co., Ltd., Japan) for phase binning. An abdominal imaging protocol was applied with a voxel size of 0.97 × 0.97 × 1.5 ${\mathrm{mm}}^{3}$.

The 4DMRI was acquired on a 1.5 T Magnetom Sola BioMatrix at HIT, using a T1_STARVIBE sequence with 8 respiratory bins with a voxel size of 1.67 × 1.67 × 1.7 ${\mathrm{mm}}^{3}$. Both 4D image sets were analyzed using the 3D Slicer software (Surgical Planning Laboratory (SPL), United States).

#### 2.4.4 Surface‐guided imaging

**FIGURE 4:**
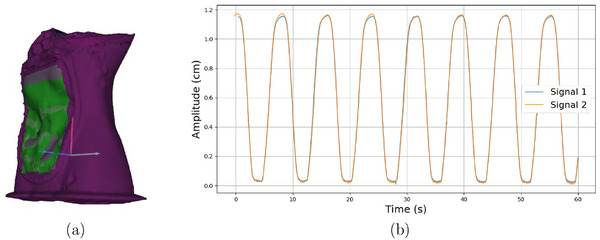
(a) 3D surface model acquired by AlignRT. The purple color presents the DICOM reference surface, gray the selected ROI and green indicates the current position of the phantom surface. (b) Reproducibility of the breathing signals acquired on two different days. ROI, region of interest.

The aim of this experiment was to determine whether the abdominal motion can be monitored on the phantom surface. For this purpose, the surface monitoring system AlignRT (VisionRT Ltd, London, UK) at HIT was used.^23^ To minimize reflections and signal distortions, a matting spray was applied to the surface of the abdomen. Further, an additional CT scan of the phantom was acquired on Definition Flash CT (Siemens Healthineers) with 120 kV, 255 mA and a voxel size (0.976 × 0.976 × 3) ${\mathrm{mm}}^{3}$. Using the RayStation treatment planning system (TPS) version 8.0.1.10 (RaySearch Laboratories, Stockholm, Sweden), the external surface of the abdomen was contoured on the CT. The lower HU thresholds used for contouring ranged from ‐250 to ‐350 HU, and the upper thresholds ranged from ‐150 to 1877 HU. This contoured surface, used as a reference in AlignRT, is called the DICOM reference surface. The phantom was initially positioned on the treatment table using three Beekley markers attached to the surface of the phantom. A ROI was then defined on the surface of the upper abdomen. AlignRT was used to monitor the phantom's motion relative to the reference surface. The current abdominal surface was compared with the DICOM reference surface within a user‐defined ROI, assessing motion across six degrees of freedom (6 DOF). To evaluate the reproducibility of surface motion, an external respiratory monitoring system, SimRT (VisionRT, London, UK), was installed in the CT room (Figure 4) and operated with the phantom in motion mode II (Figure 2). The respiratory parameters used during this procedure were identical to those employed for the 4DCT acquisition (Section 2.4.3). The reproducibility of the recorded breathing signals was assessed on two different days, and the Pearson correlation coefficient was calculated to quantify the consistency of the signals.

### 2.5 Dosimetric properties

**FIGURE 5:**
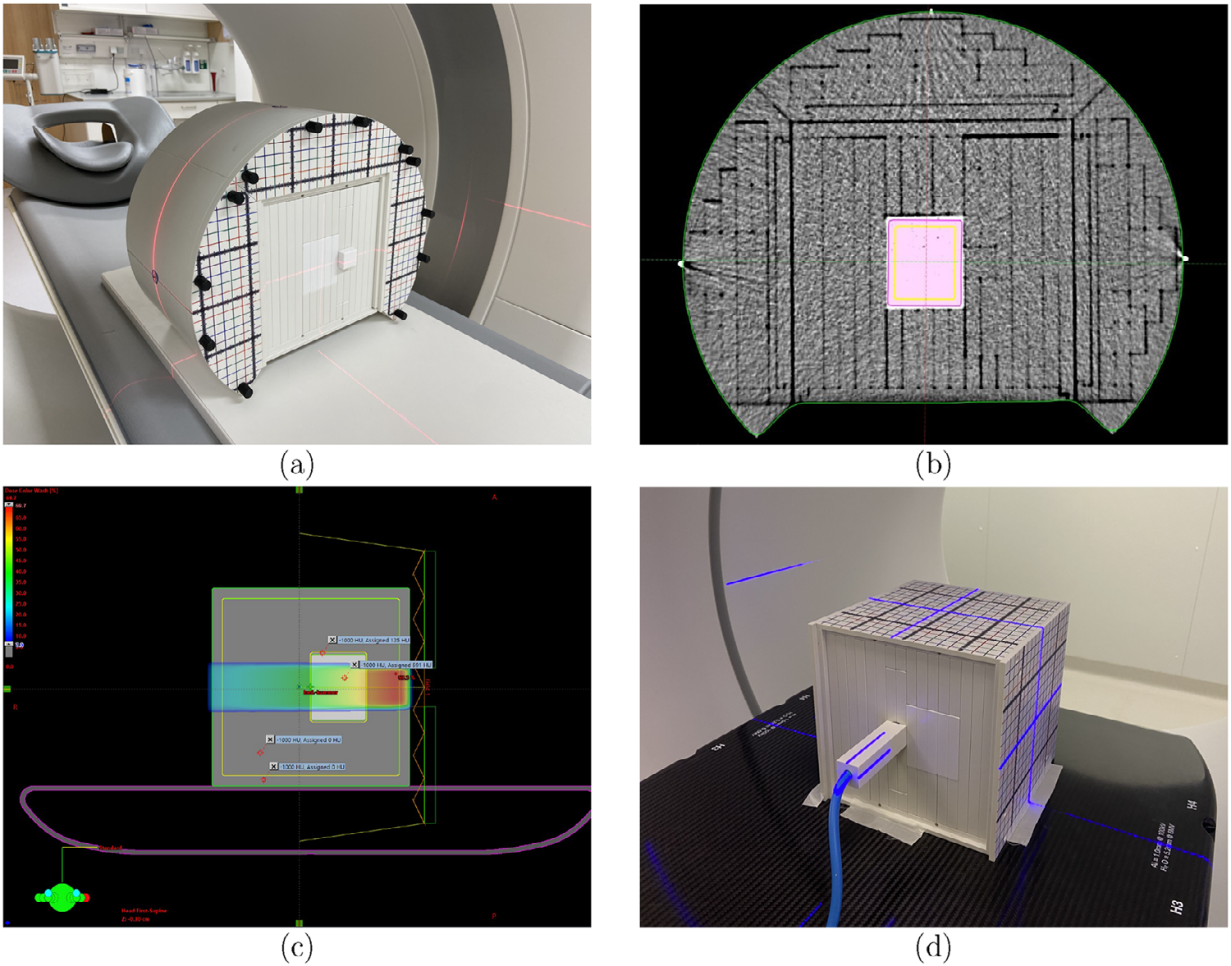
(a) Setup of the EASY CUBE at the CT at DKFZ, with a sample material positioned at the isocenter of the phantom. (b) Segmented CT scan of the ”Silicone Type 6” sample. (c) TP with a single horizontal beam, presenting bone (Gypsum) in light gray and solid water (RW3) in dark gray. The bony area is surrounded by a 1 mm layer of the container material (VeroClear). (d) Experimental setup for the dose measurement at the ETHOS linear accelerator with the ionization chamber positioned behind the material sample (IC marked by the blue laser). CT, computed tomography; DKFZ, German Cancer Research Center.

To investigate the dosimetric properties of the materials constituting BRaVIDA and to make the phantom applicable to both photon and particle quality assurance (QA) measurements, the relative electron density (RED) and the stopping power ratio (SPR) relative to water were measured. This was necessary, since the phantom includes materials that are not accounted for in the Hounsfield Look‐Up Table (HLUT) in the Eclipse TPS version 16.00.00 (Varian Medical Systems, USA). To address this issue, 3D‐printed containers (VeroClear, Stratasys, Israel) with dimensions of 60 × 60 × 50 ${\mathrm{mm}}^{3}$ were filled with the different materials and placed at the center of a water‐equivalent phantom (Easy Cube, LAP GmbH Laser Application, Lüneburg, Germany) (Figure 5). For each sample, a CT scan of the phantom was acquired with the abdomen protocol (120 kV, 300 mA, voxel size (0.97 x 0.97 x 1) ${\mathrm{mm}}^{3}$ to determine the HU values. RED and SPR were determined from a dual‐energy CT (DECT) (Somatom Confidence, Siemens, Erlangen), using voltages of 80 and 140 kV and a voxel size of (0.97 x 0.97 x 1.5) ${\mathrm{mm}}^{3}$. RED and SPR were calculated by the syngo.via version VB605_HF02 the Rho/Z software (Siemens Healthcare, Forchheim, Germany) and evaluated in the Eclipse‐TPS using a ROI of the segmented region as shown in Figure 5b.

### 2.6 Attenuation

To evaluate whether the TPS correctly predicts the attenuation of the phantom materials in a flattening filter‐free 6 MV photon beam, an Eclipse treatment plan with a single beam (gantry $90^{\circ }$, field size (4 × 4) 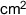) was generated based on the CTs of the materials in the Easy Cube phantom. To accurately reflect the geometric extensions of the materials and the container, synthetic contours were defined and the average HU values were assigned according to the results determined in the previous CT measurements (Section 2.4.1). From the dose calculation, the attenuation of the materials in the container relative to water was determined behind the materials and compared with the respective charge ratio obtained by using a 0.125 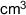 semiflex ionization chamber (TM31002‐1244, PTW Dosimetry, Freiburg, Germany). For each material, 3 measurements were performed using 100 monitor units (MU), except for the water sample, where 5 measurements were performed at the beginning and the end of the experiment (Figure 5).

## 3 RESULTS

### 3.1 Image contrast

**FIGURE 6:**
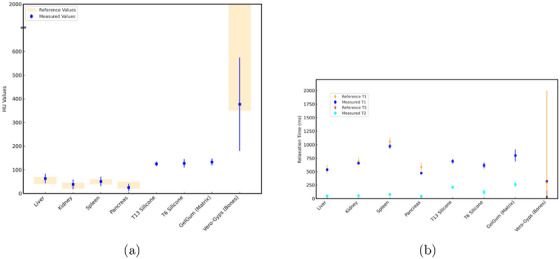
Measured versus reference HU values (a) and T1‐/T2‐relaxation times (b) for BRaVIDA's materials. The reference values are taken from Schlegel et al.^24^ No reference values are available for silicone and GelGum. BRaVIDA, Breathing Radiotherapy Visual monitoring, Imaging, and Dosimetric Anthropomorphic; HU, Hounsfield units.

The image contrast of the phantom in the CT and MRI images is demonstrated in Figure 3. The HU values of soft tissues matched the mean of reference ranges within 3% to 28% and were well within published values. T1 values were consistent with reference values, with most organs differing by less than 3% and all were within 12%. T2 values exhibited greater variability, with deviations up to 24%, the liver showing the largest difference. Reference values were not available for some organs, and bones displayed a wide range of reported values. Both HU values and relaxation times are compared with the respective reference values in Figure 6.

### 3.2 Motion monitoring

**FIGURE 7:**
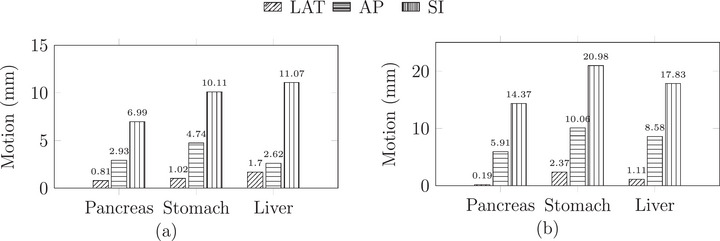
Center of mass displacement of the pancreas, stomach, and liver models measured using 4DCT (a) and 4DMRI (b), shown in the LAT, AP, and SI directions. The employed respiratory motion settings are described in Table 2. 4DCT, 4D computed tomography; 4DMRI, 4D magnetic resonance imaging; SI, superior‐inferior.

The motion amplitudes of the pancreas, stomach, and liver models measured in 4DCT and 4DMRI are displayed in Figure 7. Coronal, sagittal and transversal planes of 4DCT and 4DMRI datasets of the abdomen and the lung together with a demonstration of the phantom surface motion, are provided as videos in the supplementary material.

The use of AlignRT enabled capturing and recording of positional deviations of the phantom's surface relative to the DICOM reference surface from TP across 6 DOF, as shown in Figure 4a. To assess the reproducibility of the motion signal, SimRT was used to record the breathing signals on two separate days under arbitrarily selected but identical breathing motion settings. In this scenario, the breathing signal was generated exclusively by the diaphragm stamp with an amplitude (A) of 25 mm, resulting in a motion amplitude of 12 mm at the defined patch on the phantom surface. Figure 4b presents the recorded signals, aligned at their first maximum to highlight reproducibility. The two breathing traces exhibit a strong correlation, with a Pearson correlation coefficient of R = 0.844. The comparison of the two captured signals evaluated over a period of 60 s showed a peak‐to‐peak amplitude difference of 3.5%, a temporal shift of approximately 120 ms, and a root‐mean‐square error (RMSE) between the two signal curves of 4.2 mm.

### 3.3 RED and SPR

Based on measurements in the EASY CUBE Phantom with sample materials (Section 2.6), the RED and SPR values of the phantom materials, as determined from DECT measurements, are presented in Table 3 in comparison with the reference values, alongside the respective reference values. Maximum deviations of up to 7% in RED and 9% in SPR were observed. Taking into account the relative proportions of each material in the BRaVIDA phantom, these measurements were used as reference values for adapting the TPS.

**TABLE 3:** Measured mean RED and SPR with their corresponding standard deviations for various materials.

		RED			SPR		
Organ models	HU measured	Measured	Ref.	Dev. [%]	Measured	Ref.	Dev. [%]
Water	0.31 ± 19.80	1.00 ± 0.01	1.00	−0.1	1.00 ± 0.01	1.01	−0.6
Liver	63.13 ± 20.04	1.03 ± 0.01	1.07	−3.3	1.03 ± 0.00	1.06	−2.9
Kidney	38.31 ± 20.70	1.02 ± 0.01	1.02	0.4	1.02 ± 0.00	1.03	−1.8
Spleen	50.49 ± 19.93	1.02 ± 0.01	1.04	−1.9	1.02 ± 0.00	1.05	−2.7
Pancreas	23.05 ± 20.30	1.01 ± 0.01	1.01	0.6	1.01 ± 0.00	1.02	−0.5
Vero‐Gyps (Bone)	591.46 ± 146.24	1.27 ± 0.12	1.34	−4.8	1.22 ± 0.10	1.31	−7.2
Silicone T6	181.96 ± 23.14	1.04 ± 0.01	1.08	−4.1	1.02 ± 0.00	1.11	−7.4
Silicone T13	128.85 ± 20.82	1.00 ± 0.01	1.08	−7.3	0.98 ± 0.00	1.08	−8.9
GelGum (Matrix)	158.09 ± 21.57	1.02 ± 0.01	1.08	−5.4	1.02 ± 0.00	1.10	−7.2
Solid Cube (Veroclear)	124.58 ± 20.99	1.15 ± 0.01	1.07	7.1	1.17 ± 0.00	1.08	8.6

*Note*: For comparison, the respective reference values (Ref.) as provided by the TPS Eclipse and the deviations from measurements (Dev.) are displayed. In addition, the measured HU values are shown.

Abbreviations: HU, Hounsfield units; RED, relative electron density; SPR, stopping power ratio; TPS, treatment planning system.

### 3.4 Attenuation

To assess the accuracy of the Eclipse‐TPS in predicting the photon attenuation of the phantom materials, the attenuation of the materials relative to water was measured behind each sample in the Easy Cube phantom and was compared with calculations of the TPS. The attenuation of all materials relative to water was within 1.5%, as shown in Table 4.

**TABLE 4:** Attenuation of the materials relative to water (mean ± SD) measured with an IC compared to values calculated by the TPS Eclipse.

	Attenuation of material relative to water		
Organ models	Measured	Calculated	Deviation [%]
Liver	0.9924±0.0018	0.9858	−0.7
Kidney	0.9923±0.0018	0.9923	0.0
Spleen	0.9942±0.0018	0.9897	−0.5
Pancreas	0.9974±0.0018	0.9961	−0.1
Bone (Gypsum)	0.9298±0.0004	0.9278	−0.2
Silicone T6	0.9900±0.0004	0.9807	−0.9
Silicone T13	0.9980±0.0005	0.9832	−1.5
GelGum	0.9933±0.0018	0.9820	−1.1
Air	1.2003±0.0005	1.2036	+0.3
Veroclear	0.9716±0.0017	0.9832	+1.2

Abbreviations: IC, onization chambers; TPS, treatment planning system.

## 4 DISCUSSION

Several phantoms have been introduced to validate advanced radiotherapy workflows. However, most of these developments address only selected aspects of the treatment chain and are therefore limited in their scope of application.^25^ Common restrictions are the lack of anthropomorphic organ stuctures,^9^, ^10^, ^19^ respiratory motion,^17^ multimodal imaging contrast,^13^, ^17^, ^18^ or precise dosimetric capabilities, while at the same time maintaining compatibility with SGRT applications.^12^, ^18^ The **BRaVIDA** phantom avoids these limitations and offers new applications in RT and radiology. It incorporates 12 organs in the abdomen and thorax, along with tumors, and provides realistic tissue contrasts in both CT and MRI. This is important when testing adaptive RT workflows, which are intrinsically connected to guidance by radiological or surface images. With anthropomorphic image contrast in CT and MRI particularly for the liver, pancreas, kidneys, and spleen, it becomes possible to include also the imaging procedure in workflow tests. The deformability of BRaVIDA allows simulating interfractional anatomical changes (e.g., by changing the air or water volumes in the lungs or colon/stomach, respectively to simulate a pleural effusion in the lung or the peristalsis of the gastrointestinal organs). These deformable structures of BRaVIDA may also be employed for testing automated segmentation, deformable image registration or the creation of synthetic datasets such as synthetic CT as required in MR‐only RT. Using predefined breathing patterns, intrafractional motion can additionally be simulated, which allows testing of motion compensation techniques such as beam gating or tracking. Furthermore, as the thoracic part of the phantom is removable for insertion of the stamp system, the lung model could technically be replaced with an updated 3D‐printed version containing embedded lesions or additional anatomical structures.

The HS used to simulate organ motion supports arbitrary respiratory patterns, including uncontrolled movements, such as coughing or sneezing. However, the reproducibility of these motion patterns may be limited by the presence of air bubbles in the HS and as a result also the maximum achievable organ movements may be limited. Enhancing the linearity of the HS by reducing air in the system could increase motion amplitude.

Shore tests on various GelGum (Matrix) mixtures demonstrated that adjusting the ratio of the consisting silicone components A (blue) and B (white) reduces the Shore hardness with respect to the 1:1 standard ratio. This adjustment also has the potential to increase the motion amplitude without significantly affecting HU values in CT. This can be explained by the fact that component A (blue) has a HU value of 136, while that of component B (white) is only slightly lower (126 HU). In contrast, T1‐ and T2‐relaxation times changed from 725 ms and 174 ms to 745 ms and 403 ms, respectively.

Besides for classical measurements in radiology and RT, the phantom might also be applied to test the robustness and generalizability of AI models (e.g., used for segmentation, registration or motion prediction), as the phantom essentially allows for an unlimited number of intentional and random motion patterns, needed to generate training and validation datasets. A limitation of such studies may be, however, that there are still differences in image and motion patterns between the BRaVIDA phantom and patients that may limit the direct application of a BRaVIDA‐trained AI‐algorithm in the clinic.

In addition to investigating imaging aspects, the phantom may be equipped with detectors, such as films or ionization chambers, to measure the dose to a tumor or to organs at risk delivered by photon beams. For this, it is important that the attenuation of photons in the different phantom materials is accurately predicted by the TPS. This has been validated and the results show that the dose deviations between measurement and TPS calculation using the AAA algorithm are less than 2 % for all materials. Therefore, the BRaVIDA phantom gives reliable dosimetric results and equipped with detectors, the phantom may therefore be used for comprehensive dosimetric E2E tests in photon RT workflows including inter‐ and intrafractional motion as well as static and 4D imaging protocols. For ion beams, an adjustment of the SPR in the HLUT with the values measured in this study is necessary to assure a correct range calculation by the TPS. After this is done, the BRaVIDA phantom is expected to be applicable for ion beams as well. The phantom demonstrated a stable performance over the time of measurements (14 months), supporting its suitability for reproducible dosimetric and imaging tests. Nevertheless, long‐term stability and durability under repeated use have not yet been assessed and needs to be systematically investigated in future studies to confirm its long‐term reliability for clinical and research applications.

## 5 CONCLUSION

This study presents a versatile, MRI‐compatible phantom exhibiting anthropomorphic image contrast, inter‐ and intrafractional motion as well as radiation attenuation in photon beams, and equipping the phantom with detectors allows for full end‐to‐end‐tests in adaptive image‐ and surface‐guided photon RT workflows. For ion beams, the SPR values in the TPS have to be adapted to the measured values to assure correct range calculation in the phantom. The phantom presents a valuable and accurate tool for clinical and research applications in adaptive RT.

## CONFLICT OF INTEREST STATEMENT

The authors declare no conflicts of interest.

## Supporting information



Supporting Information
