# Supplementary figures and images for: A dynamic anthropomorphic phantom for end‐to‐end testing in image‐ and surface‐guided adaptive radiotherapy
Source: Med Phys. 2025 Nov 3;52(11):e70107. doi: 10.1002/mp.70107 (PMC12583891; doi:10.1002/mp.70107)

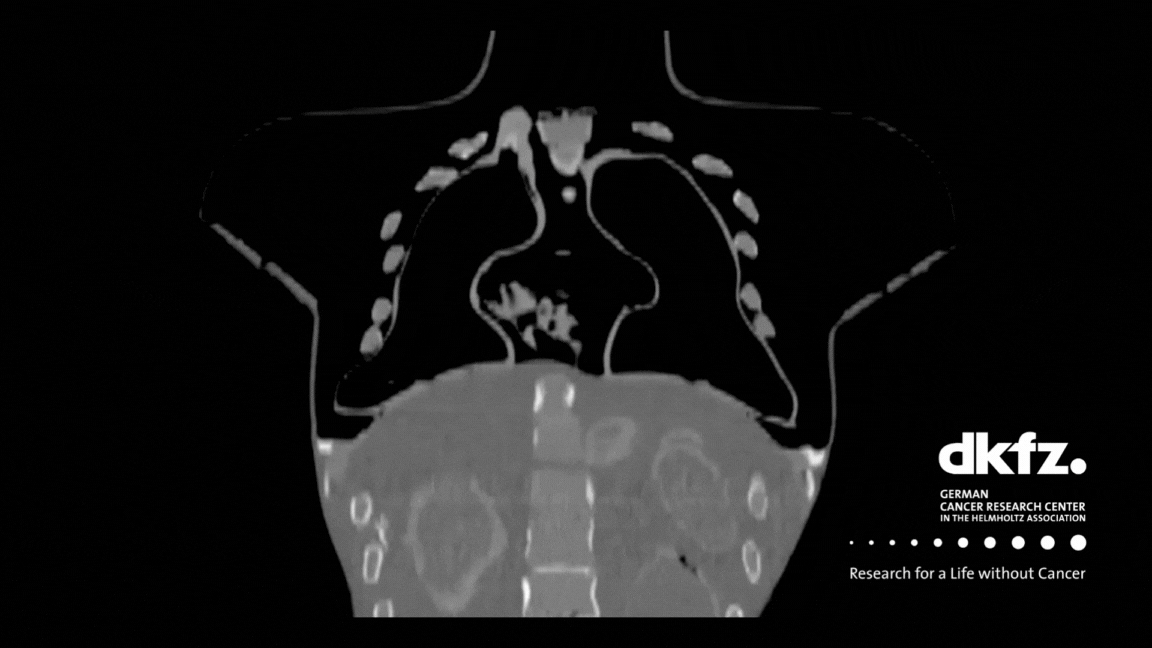

Supplement: Supplementary file 1 — Supporting Information [file MP-52-0-s001.zip › 4DCBCT Lung/Coronal Lungs.gif]

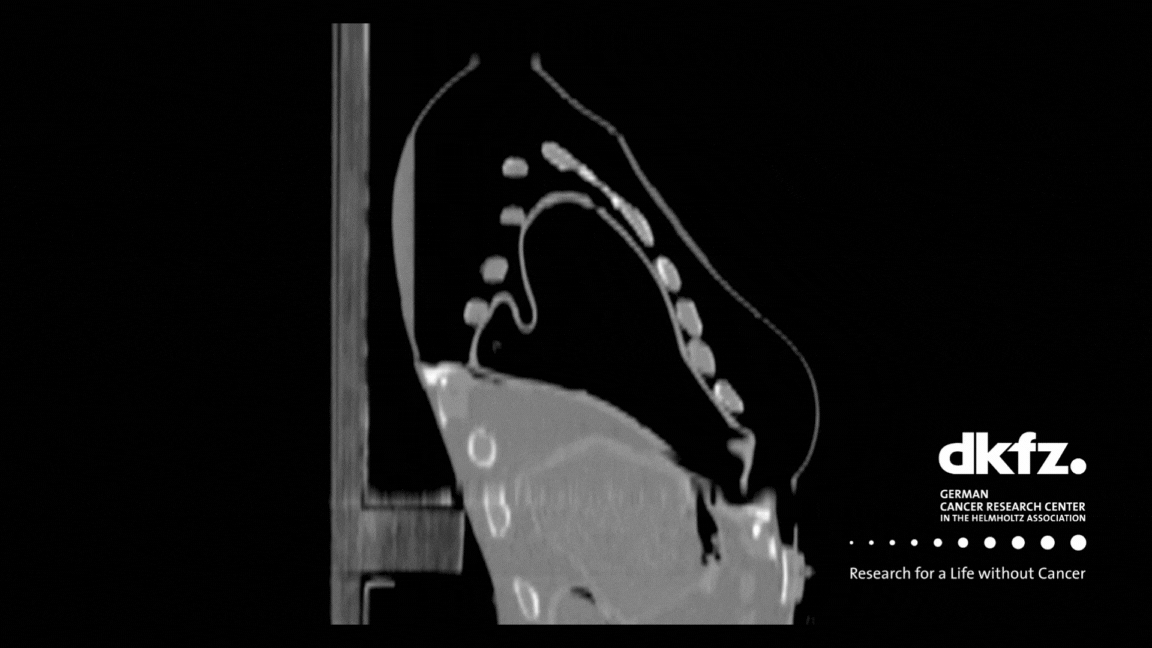

Supplement: Supplementary file 1 — Supporting Information [file MP-52-0-s001.zip › 4DCBCT Lung/Sagital Lungs.gif]

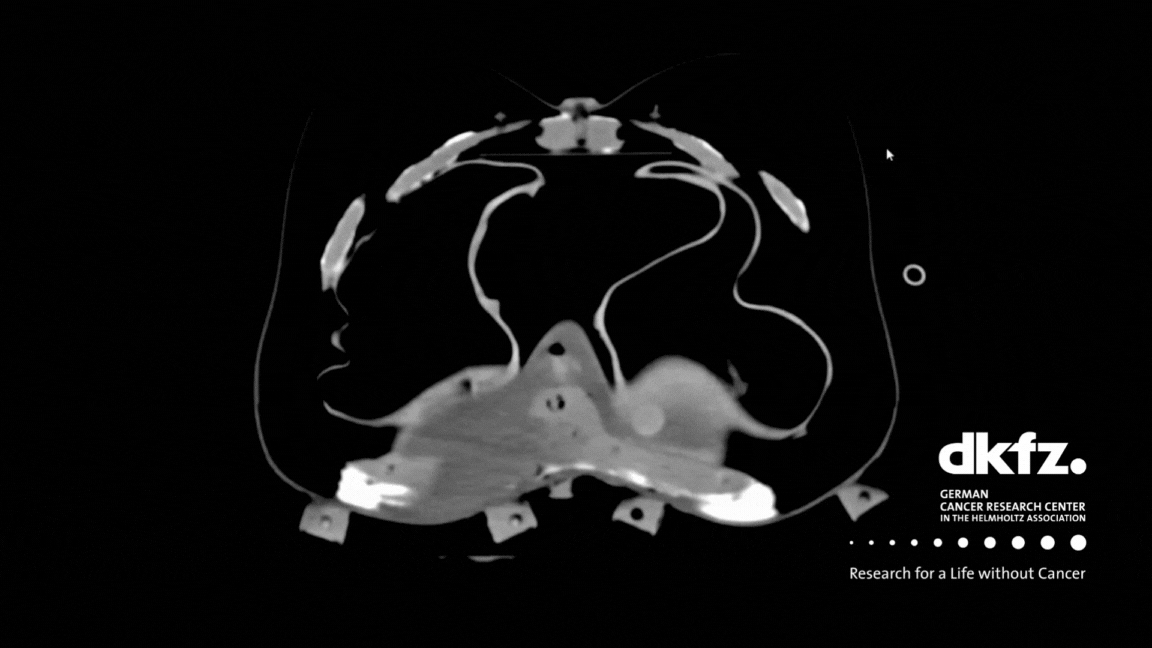

Supplement: Supplementary file 1 — Supporting Information [file MP-52-0-s001.zip › 4DCBCT Lung/Transversal Lungs.gif]

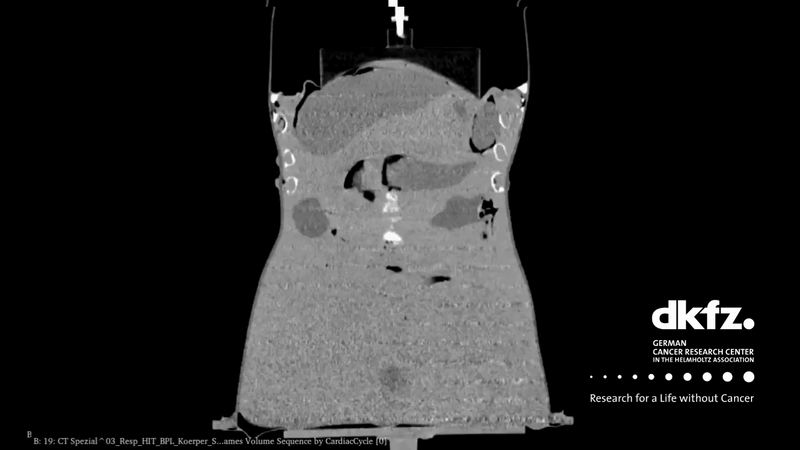

Supplement: Supplementary file 1 — Supporting Information [file MP-52-0-s001.zip › 4DCT/Coronal-ezgif.com-video-to-gif-converter (1).gif]

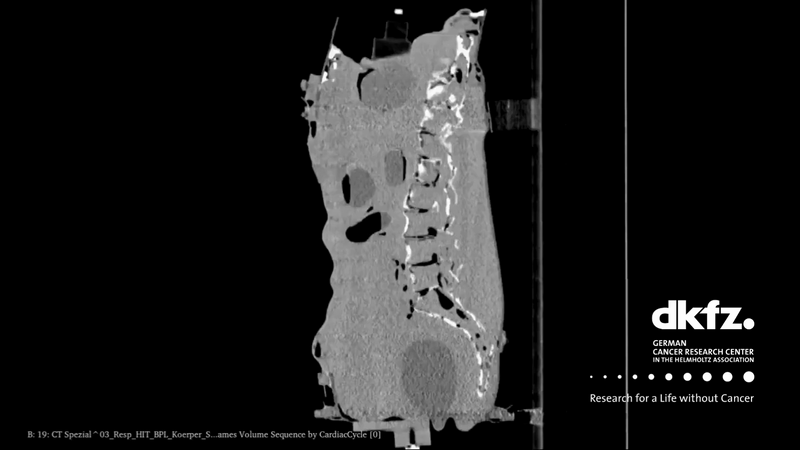

Supplement: Supplementary file 1 — Supporting Information [file MP-52-0-s001.zip › 4DCT/Sagital-ezgif.com-video-to-gif-converter.gif]

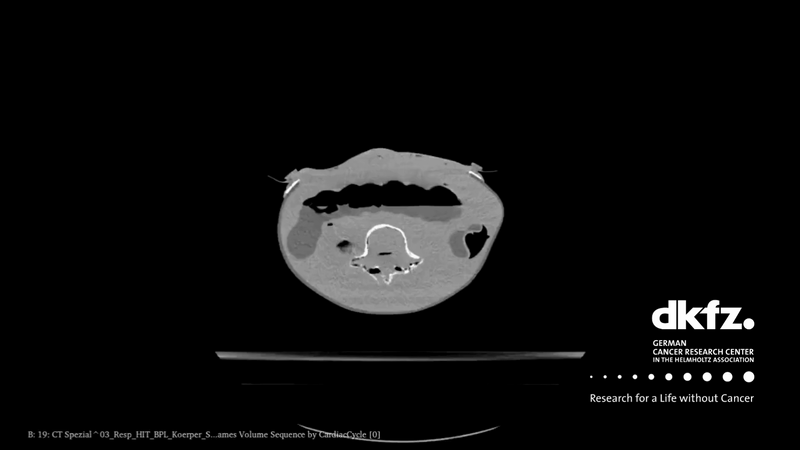

Supplement: Supplementary file 1 — Supporting Information [file MP-52-0-s001.zip › 4DCT/Transversal-ezgif.com-video-to-gif-converter.gif]

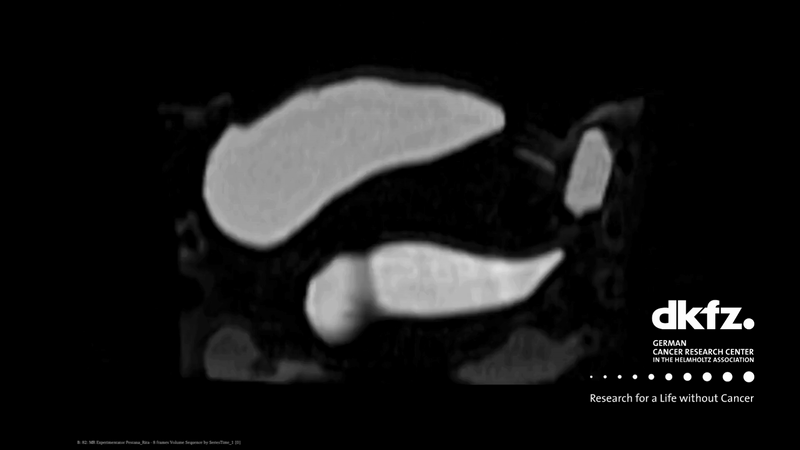

Supplement: Supplementary file 1 — Supporting Information [file MP-52-0-s001.zip › 4DMRI/CoronalWithLogo-ezgif.com-video-to-gif-converter.gif]

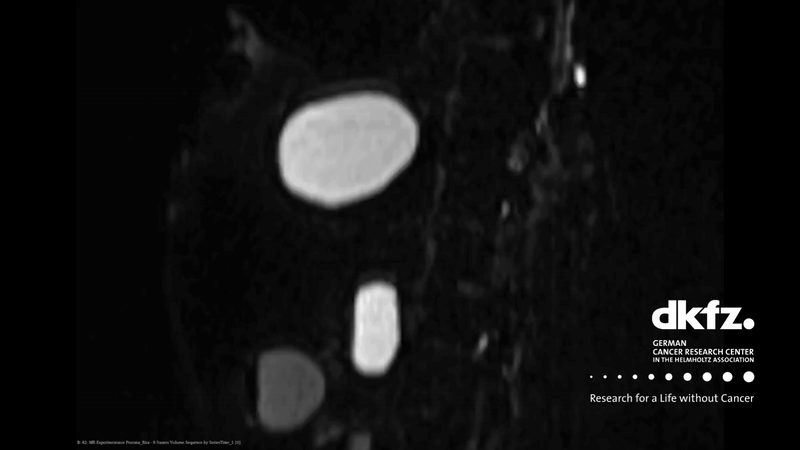

Supplement: Supplementary file 1 — Supporting Information [file MP-52-0-s001.zip › 4DMRI/Sagital-ezgif.com-video-to-gif-converter (1).gif]

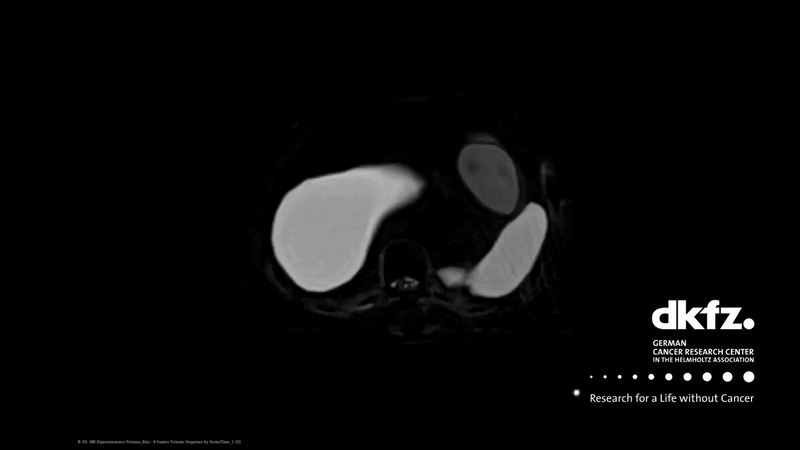

Supplement: Supplementary file 1 — Supporting Information [file MP-52-0-s001.zip › 4DMRI/Transversal.com-video-to-gif-converter (1).gif]

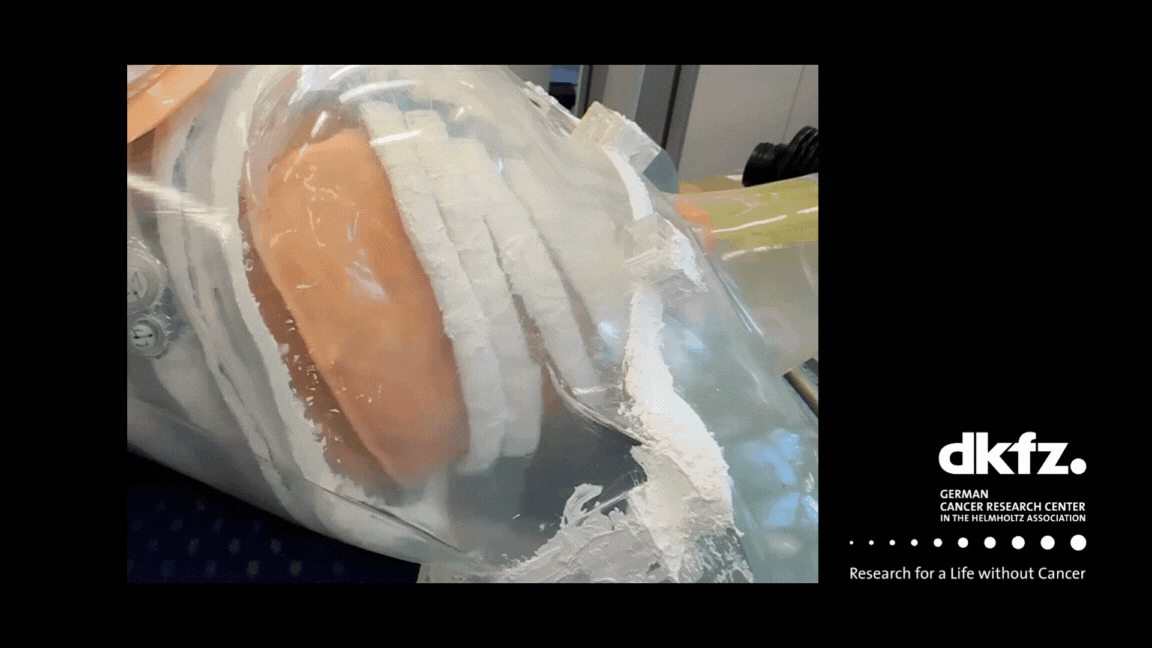

Supplement: Supplementary file 1 — Supporting Information [file MP-52-0-s001.zip › BRaVIDA Surface/Abdomen and Lung Motion.gif]
